# Advances and challenges in endoscopy training: A mixed methods study among endoscopy trainers in the Netherlands

**DOI:** 10.1055/a-2370-5812

**Published:** 2024-09-09

**Authors:** Robert A Mousset, Agnes Diemers, Wouter H de Vos tot Nederveen Cappel, Jean-Pierre E.N. Pierie, Alexandra M.J. Langers, Paul L.P. Brand

**Affiliations:** 1 10173 Lifelong Learning, Education and Assessment Research Network (LEARN), University Medical Centre Groningen, Groningen, Netherlands; 2 8772 Gastroenterology and Hepatology, Isala Zwolle, Zwolle, Netherlands; 3 Gastroenterology and Hepatology, Isala Hospital, Zwolle, Netherlands; 4 4480 Department of Surgery, Medisch Centrum Leeuwarden, Leeuwarden, Netherlands; 5 Gastroenterology, Leiden University Medical Center, Leiden, Netherlands; 6 8772 Department of Medical Education and Faculty Development, Isala Zwolle, Zwolle, Netherlands

**Keywords:** Endoscopy Upper GI Tract, Endoscopy Lower GI Tract, Quality and logistical aspects, Training

## Abstract

**Background and study aims** Variation between trainers in providing traditional gastrointestinal endoscopy training, in which residents learn endoscopy by doing under the supervision of endoscopy trainers, may cause differences in endoscopy competence between residents. In this study, we explored endoscopy trainers’ views on the current status and desired future best practices regarding endoscopy training.

**Methods** This mixed-methods study comprised quantitative survey data collected from 158 endoscopy trainers working in 26 gastroenterology teaching hospitals in the Netherlands and semi-structured interviews with 15 gastroenterology residency (associate) program directors (PDs). Survey data were analyzed using descriptive statistics and interview results with thematic analysis.

**Results** There was considerable variability in endoscopy training practices between teaching hospitals in the structure of endoscopy supervision, criteria used to determine the level of supervision, and participation of trainers in endoscopy teaching courses. Interview participants agreed that an endoscopy training supervisor requires several teaching strategies, highlighting the importance of formal education in endoscopy teaching. Interview participants perceived self-regulated learning strategies as essential for residents to learn endoscopy effectively. The perceived main barriers to effective supervision were a lack of time and heavy workload. Participants expressed the desire for more standardization in endoscopy training programs between teaching hospitals.

**Conclusions** Considerable variability in endoscopy training practices between gastroenterology teaching hospitals was identified. Formal education on endoscopy teaching, promotion of self-regulated learning, and standardization of endoscopy training programs and supervision practices have the potential to improve future endoscopy training.

## Abstract (de)

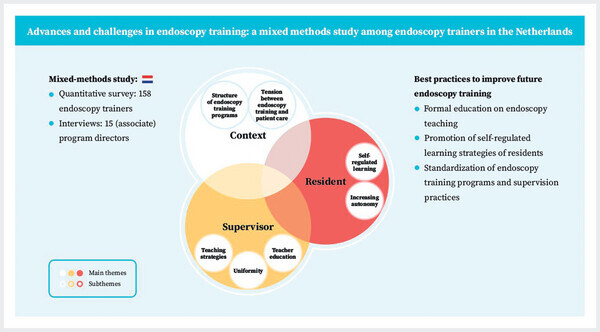

## Introduction

Training in gastrointestinal endoscopy is a cornerstone of gastroenterology residency programs. Traditionally, endoscopy has been taught following the apprenticeship model, in which residents learn endoscopy through hands-on experience under the supervision of different trainers 1 2 .

This method of endoscopy training is challenging for several reasons. First, trainers must explicitly verbalize complex cognitive and psychomotor skills and communicate in an understandable manner to residents, without having control of the endoscope 3 4 . Second, trainers must balance the learning needs of residents while ensuring patient safety, procedure time, and quality 3 . Third, the increasing focus on endoscopy quality and safety has led to a growing emphasis on resident competence. Trainers are required to use competency-based assessment, such as Entrustable Professional Activities (EPAs) 5 , to optimize individualized endoscopy learning and ensure resident competence prior to independent practice 6 . Finally, unlike in the United Kingdom (UK), in most countries, formal education about how to teach endoscopy training effectively is not standard 2 7 8 . This may lead to inconsistencies that create confusion for residents 9 10 .

In 2017, the European Section and Board of Gastroenterology and Hepatology introduced the European curriculum of Gastroenterology and Hepatology training in order to harmonize and set standards in gastroenterology education, including endoscopy training 11 . Despite this, studies among gastroenterology residents revealed considerable variability, both between and within countries, regarding the type and number of endoscopies performed during residency training, resident participation in a preclinical endoscopy course, exposure to simulator training, criteria used to determine the level of supervision, and supervisor uniformity 10 12 . These studies, however, did not cover the trainers’ perspectives, which is essential to develop a robust understanding of the current educational process and to explore future best practices.

In the Netherlands, the duration of gastroenterology residency varies from 65 to 72 months, depending on residents’ individual competencies 13 . Following 20 months of internal medicine training, residents learn to perform endoscopies on patients under the direct supervision of trainers (EPA level 2) in their first year. After achieving a specified level of proficiency, residents are declared competent to perform endoscopies under indirect supervision (EPA level 3) or with supervision on request (EPA level 4). Direct observations of procedural skills (DOPS) of individual endoscopic procedures are used to assess resident competency and provide structured feedback 13 14 . Although the training schedules of individual residents may differ, most residents complete their first 2 years of gastroenterology residency in a general teaching hospital and the final 2 years in a university hospital. Whether there are training differences between university hospitals and general teaching hospitals has not been studied to date.

To explore the aforementioned gaps in the literature, we conducted a mixed-methods study that aimed to evaluate and compare endoscopy trainers’ views about the current status of and desired future best practices for endoscopy training.

## Materials and methods

### Study design

This mixed-methods study was conducted in all 26 gastroenterology teaching hospitals in the eight training regions in the Netherlands. Each training region consists of one university hospital and two or three affiliated general teaching hospitals. The study comprised a quantitative survey, intended to assess the current status of gastrointestinal endoscopy training practices from the perspective of endoscopy trainers, followed by semistructured interviews. The survey results provided input for the topic guide of the interview study. The interviews aimed to elaborate upon the quantitative findings, with a particular emphasis on perceived strengths, barriers, and opportunities regarding endoscopy training.

### Participants and procedure

#### Online survey

Recruitment for the online survey started with an introductory e-mail to the program directors (PDs) of the 26 gastroenterology teaching hospitals, outlining the study aims and methods and requesting their participation in the study. After all PDs gave their consent, a survey hyperlink was sent to all 306 endoscopy trainers (185 in general teaching hospitals and 121 in university hospitals) in January 2022 with two subsequent e-mail reminders. Written informed consent was obtained from all participants.

The 32-item survey ( **Appendix 1** ) was developed by the research team and largely based on our previously performed survey among gastroenterology residents 10 . Questions were open- and closed-ended and were presented as single-answer, multiple choice, 5-point Likert scale (ranging from 1 – “strongly disagree” to 5 – “strongly agree”), and free-text. Questionnaires were collected using the secure web application REDCap (Research Electronic Data Capture).

#### Semistructured interviews

For the semistructured interviews, we purposely invited one (associate) PD working in a university hospital and one (associate) PD working in a general teaching hospital from each training region 15 . All participants were experienced endoscopy trainers. Participants were invited by e-mail, which stated the purpose of the study and provided assurance about the anonymity and confidentiality of all data. During the invitation period, two university hospitals formally merged, which reduced the number of teaching hospitals in the Netherlands from 26 to 25 and the number of training regions from eight to seven. Therefore, we decided to enroll 15 PDs: seven working in university hospitals and eight working in general teaching hospitals. All invited participants consented to participate. After two pilot interviews with members of the research team through which the interview guide was refined, RM conducted semistructured interviews with all participants between May 1, 2023 and July 31, 2023. Oral (recorded) and written informed consent was obtained prior to the interviews. The interviews were conducted in-person or online, depending on participant preference, and guided by a semistructured interview guide ( **Appendix 2** ). The interviews lasted 30 to 45 minutes and were tape recorded and transcribed verbatim using Amberscript 16 . Member checks were conducted afterwards. Minor feedback was given by one participant. Iterative data collection and analysis occurred simultaneously, allowing minor adaptations to the interview guide when necessary.

#### Data analysis

Quantitative data from the online survey were analyzed using SPSS Statistics version 26. Differences between gastroenterologists’ responses from university and general teaching hospitals were analyzed using Pearson’s χ ^2^ tests and T-tests. All *P* values were two-sided, with *P* < 0.05 considered statistically significant.

Interview data were analyzed using Braun and Clarke’s approach to thematic analysis 17 . After familiarizing themselves with the data, two authors (RM and AD) inductively coded the first three interview transcripts independently and compared all codes. Inconsistencies were discussed until consensus was reached. Once finalized, each subsequent interview was inductively coded by RM and a random selection of all coded transcripts was critically examined by AD. After reaching consensus on code inconsistencies, RM recoded all transcripts using the finalized code tree, and RM and AD consecutively sorted the identified codes into initial themes. These were reviewed and discussed by the research team until consensus was reached on main themes and sub-themes. Qualitative data analysis was performed using ATLAS.ti version 23 18 .

### Ethics approval

The study was reviewed and approved by the Medical Ethical Committee of Isala Hospital, Zwolle, the Netherlands (study number: 210117).

## Results

### Background characteristics

A total of 158 endoscopy trainers completed the online survey (52% response rate). After excluding four respondents who did not disclose their hospital type, our study sample comprised 154 respondents: 115 (75%) from general teaching hospitals and 39 (25%) from university hospitals. All 26 teaching hospitals were represented. We performed in-depth interviews with 11 gastroenterology residency PDs and four associate PDs. Background characteristics for both the survey and interview participants are summarized in Table 1 .

**Table:** **Table 1** Characteristics of survey respondents and interview participants.

	All survey respondents	General teaching hospital survey respondents	University hospital survey respondents	Interview participants
	(N = 154)	(N = 115)	(N = 39)	(N = 15)
Mean age, years (SD)	47.1 (8.5)	47.2 (8.2)	46.9 (9.3)	49.1 (7.0)
Female, N (%)	64 (42)*	46 (40)*	18 (46)	7 (47)
Mean endoscopy trainer experience, years (SD)	10.5 (6.5)	10.4 (6.1)	(7.7)	13.1 (5.7)
Role				
Program director, N (%)				11 (73)
Associate program director, N (%)				4 (27)
N, number of participants; SD, standard deviation. *Missing data.				

### Online survey

**Fig. 1:**
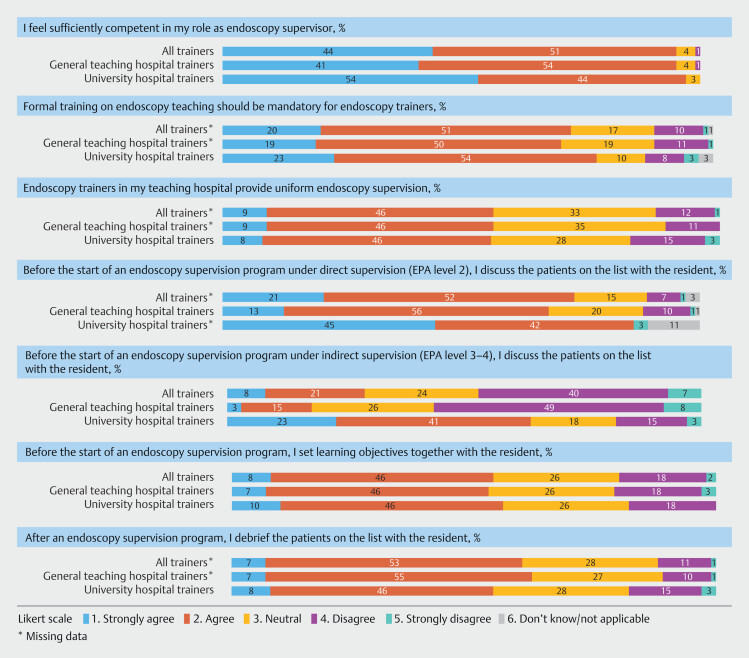
Proportion of trainer responses to Likert score questions regarding endoscopy supervision.

Endoscopy supervision practices in university hospitals and general teaching hospitals differed considerably ( Table 2 ). University hospital gastroenterologists reported more endoscopy supervision programs but fewer residents supervised at the same time than those in general teaching hospitals. Gastroenterologists in general teaching hospitals were more likely to have their own endoscopy program during indirect supervision of more experienced residents (EPA level 3/4) than those in university hospitals. Almost all respondents (96%) perceived themselves to be competent in training residents in gastrointestinal endoscopy ( Fig. 1 ). Forty-nine percent (19/39) of university hospital gastroenterologists had participated in an endoscopy teaching course, compared with 25% (29/115) of the gastroenterologists from general teaching hospitals ( *P* = 0.006). Most respondents (71%) (strongly) agreed that participation in an endoscopy teaching course should be mandatory for endoscopy trainers. Fifty-five percent (83/154) reported uniformity in endoscopy teaching methods between different trainers in their teaching hospital. Criteria used to determine the level of supervision differed between teaching hospitals. The transition from direct (EPA level 2) to indirect supervision (EPA level 3) was based on competence assessment according to 89 respondents (58%) and on a predefined time period according to 37 respondents (24%). The predefined direct supervision period varied between teaching hospitals (median 12 weeks; interquartile range [IQR] 8–16 weeks). Seven respondents (4%) reported that the direct supervision period was based on threshold numbers (median 65 procedures; IQR 50–170 procedures). Twenty-one respondents (14%) did not know the criteria used for determining the level of supervision.

**Table:** **Table 2** Comparison of endoscopy training characteristics between respondents in university hospitals and general teaching hospitals.

	All survey respondents	General teaching hospital survey respondents	University hospital survey respondents	*P* value
	(N = 154)	(N = 115)	(N = 39)	
Number of supervision programs, half days a week (SD)	2.1 (1.2)	1.8 (0.9)	3.0 (1.4)	**< 0.001**
Number of residents per supervision program (SD)	1.7 (1.0)	1.8 (1.1)	1.4 (0.6)	**.028**
I supervise residents under direct supervision, N (%)	137 (90)*	112 (98)*	25 (64)	**< 0.001**
I supervise residents under indirect supervision, N (%)	151 (99)*	113 (99)*	38 (97)	0.423
I have my own endoscopy program during indirect supervision, N (%)	69 (46)*	62 (55)*	7 (18)*	**< 0.001**
Having participated in an endoscopy teaching course, N (%)	48 (31)	29 (25)	19 (49)	**0.006**
SD, standard deviation. Statistics used were the T-test and χ ^2^ -test. *Missing data				

### Interviews

**Fig. 2:**
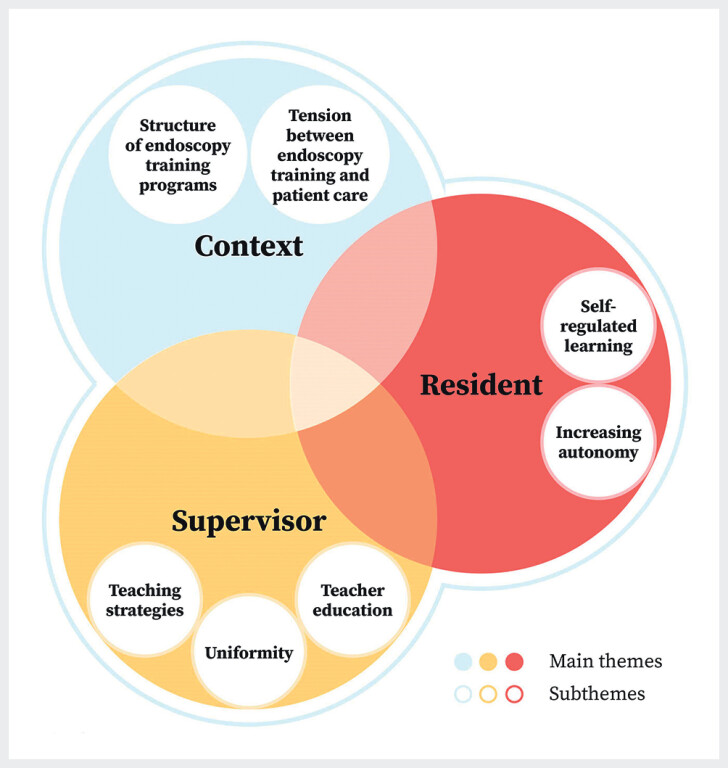
Perceived strengths, barriers, and opportunities regarding endoscopy training.

The interviewed PDs considered future endoscopy training as a dynamic task that demands specific skills and shared commitment from supervisors and residents, embedded in an effective learning environment ( Fig. 2 ). Each of the identified main themes (supervisor, resident, context) is described in more detail, with corresponding subthemes and representative participant quotes.

#### Supervisor

PDs reported various effective teaching strategies to train residents in endoscopy. These included a discussion between supervisor and resident before and after an endoscopy training program to set and evaluate learning objectives, determination of earlier acquired endoscopy experience, use of consistent terminology, verbal instruction without taking over the endoscope, and avoidance of cognitive overload. However, due to contextual factors, such as lack of time and heavy workload, participants experienced a gap between ideal and actual supervision practices. PDs expressed a shared responsibility between supervisor and resident to improve this.

*“* I repeatedly emphasize the importance of setting learning objectives to supervisors and residents. They should discuss in advance: what are the learning objectives today?” (P9)

Most PDs proposed that endoscopy competence of residents is developed with direct supervision and extensive feedback, rather than by performing many procedures. Deconstructing endoscopic procedures into discrete component steps was considered highly effective, to provide supervisors with a structured framework for training endoscopy, analyzing endoscopy competence, and giving constructive feedback.

“I love task deconstruction, because in my opinion that is the most effective teaching method. (…) The resident focuses on one learning objective. I save time by performing the procedure partly myself, allowing us to spend more time on the learning objective.” (P23)

Most participants noted that lack of uniformity between different supervisors led to conflicting messages that create confusion for residents, especially in the first phase of endoscopy training. By contrast, some perceived variation in supervisor perspectives and approaches to promote the resident learning process. One PD proposed that teaching hospitals should have one or two experienced supervisors to train novice residents in endoscopy.

“There are so many changes in supervisors, all having their own focus. That is difficult for novice endoscopists. When they [residents] have experience, it is an advantage because they can learn from the different styles, techniques, points of attention. But at first, it is confusing.” (P8)

Formal education on effective endoscopy teaching was perceived to be very beneficial in becoming a “consciously competent trainer”, and to improve supervisor uniformity within teaching hospitals. The desire for continuous learning through follow-up supervision training was expressed by several PDs who had participated in an endoscopy training course.

“A teach-the-endoscopy-teacher course supports you to become a better teacher. What are your own pitfalls? What are your teaching styles? And how can you improve?” (P20)

#### Resident

Participants believed that self-regulated learning, the process in which residents take responsibility and initiative in diagnosing their learning needs, formulating learning objectives, and requesting supervisor assistance and procedure feedback, is important to optimize endoscopy learning.

“What certainly can be improved is that they [residents] ask for DOPS. Because I think… When they perform endoscopies themselves, they rarely ask for feedback.” (P11)

“An endoscopy supervision program that did not go well. Some residents are not able to set learning objectives…And it irritates me when a resident is not able to self-reflect.” (P20)

In clinical practice, the transition from direct (EPA level 2) to indirect endoscopy supervision (EPA level 3) for novice endoscopists was not solely based on competence assessment. Some teaching hospitals use a predefined period of direct supervision. Depending on a resident’s learning curve, the direct supervision period could be shortened or extended. One PD indicated that an extended period of direct supervision was difficult to schedule. Several PDs perceived the DOPS assessment tool, used to assess the endoscopy competence of residents, to be too extensive and time-consuming to be useful in endoscopy supervision practice. The decision about whether residents can progress to a higher EPA level is usually made during a formal staff meeting. Some teaching hospitals took the opinion of endoscopy nurses into account regarding this decision.

“We also ask the opinion of the endoscopy nurses [regarding the resident’s endoscopy performance]…The feedback [of endoscopy nurses] is valuable, because the resident behaves differently with attendance of the supervisor. For example on patient interactions.” (P5)

#### Context

PDs acknowledged the tension between endoscopy training and patient care. The main barriers to effective endoscopy supervision that were identified were lack of time and heavy workload. In some teaching hospitals, endoscopy supervisors performed multiple tasks in parallel with supervising residents, such as their own endoscopy program and supervision of medical students.

“Lack of time is the most important factor. When the patient is delayed, there is less time for supervision. In that case, the resident’s training is inferior to the care of the patient. That annoys me.” (P26)

Participants described considerable differences in the structure of endoscopy training programs between teaching hospitals and expressed a desire for more standardization in both the initial and advanced stages of residency. Although PDs universally reported that participation in an endoscopy training course is mandatory before residents start performing endoscopies on patients, access to endoscopy simulator training for further practice after completion of the mandatory course was available only in a few teaching hospitals. One participant proposed standardization of preclinical simulator training.

“We should make agreements about the extent to which a resident practices on an endoscopy simulator under supervision of a gastroenterologist before performing endoscopic procedures on patients.” (P7)

PDs stated that residents should preferably start their endoscopy training in a general teaching hospital, because of the focus on highly complex healthcare and lack of low-complexity endoscopies in university hospitals. PDs suggested limiting advanced endoscopy training in the final years of gastroenterology residency to dedicated subspecialty training programs, with exposure in both university and general teaching hospitals. They envisioned a skills-based selection for these programs, aligned to the demand for a particular endoscopic procedure to ensure adequate exposure.

“Residents are very satisfied [about the advanced subspeciality training programs]! …For example, the resident in the HPB [hepato-pancreato-biliary] training program performs all elective [esophageal] variceal band ligation procedures. The learning curve is steep.” (P15)

## Discussion

This mixed-methods study aimed to deepen our understanding of endoscopy trainers’ perspectives about the current status of and desired future best practices for gastrointestinal endoscopy training. Considerable variability in endoscopy training practices between and within teaching hospitals was found, in line with our previous study among residents 10 . To overcome the challenges faced and improve future endoscopy training, best practices were identified regarding supervisors, residents, and context.

In the interviews, PDs reported about various teaching strategies that were effective for training residents in endoscopy, including discussing learning objectives with residents before and after an endoscopy training program, providing direct supervision and feedback, and task deconstruction. These strategies reflect the deliberate practice theory of acquiring competence in complex skills 19 and have been identified as essential tools for endoscopy trainers 3 4 6 7 8 20 21 . However, survey results from this study and our previous study among residents indicate that these strategies are rarely applied in current endoscopy supervision practice 10 . This might be due to contextual factors, such as lack of time and heavy workload, lack of (shared) understanding of supervisors and residents about the purpose and relevance of supervision, or lack of teaching skills 22 . Multiple studies have suggested that formal education about endoscopy teaching and standardization of teaching methods may improve the quality of endoscopy training 2 4 6 7 21 .

Although comparative studies are lacking, it is plausible that there is more variability in teaching methods in the Netherlands, where only a minority of trainers receive formal training on endoscopy teaching, compared with countries, such as the UK, where most trainers have been trained. A recent survey study in the UK revealed that endoscopy trainers who completed a train-the-trainer (TTT) course more commonly reported setting learning objectives and completing DOPS compared with untrained trainers 8 . Survey respondents in our study perceived an endoscopy teaching course to be so useful that it should be mandatory for endoscopy trainers. In laparoscopic colorectal surgery, implementation of a TTT course improved both the teaching performance of trainers and learning curves of residents 23 . We are currently evaluating whether a train-the-colonoscopy-trainer course has similar effects on endoscopy trainers and residents.

PDs believed that residents’ self-regulated learning (SRL) strategies, such as diagnosing own learning needs, formulating learning objectives and requesting procedure feedback, can improve endoscopy learning, in line with the deliberate practice theory 19 . SRL refers to modulation of “self-generated thoughts, feelings, and actions that are planned and cyclically adapted to the attainment of personal goals” 24 . Elements of SRL demonstrated to be effective in gastrointestinal endoscopy training include colorectal polyp classification 25 and endoscopy simulator performance 26 . SRL also appeared to be feasible in patient-based colonoscopy training and was highly valued by residents 27 . Although SRL can improve complex psychomotor skills, it requires specific training and guidance 28 . Due to the Dunning-Kruger effect, incompetent learners, including novice endoscopists, have a tendency to overestimate their abilities 29 30 . Therefore, implementation and development of SRL in endoscopy training practice requires interventions to improve residents’ goal setting and reflection skills. Further, the learning environment should offer opportunities for residents to employ their SRL skills, including supportive trainers and sufficient time for supervision 31 . In spite of the paradigm shift in medical education from use of threshold numbers toward a competency-based approach 1 , our study results indicated that the level of supervision of novice endoscopists is both time- and competency-based. These findings resonate with the previously mentioned residents’ experiences 10 . Important barriers to use of competence assessment were scheduling difficulties and logistical concerns regarding the DOPS assessment tool, in agreement with the literature 6 . Future research is required to develop strategies to overcome these barriers.

Endoscopy supervision practices varied considerably between university and general teaching hospitals. Participants expressed a desire for more standardization in both the initial and advanced stages of endoscopy training. To overcome disparities in development of basic endoscopic skills of novice endoscopists, PDs suggested integrating simulator training into the national gastroenterology training curriculum. This has been shown to significantly improve novice endoscopists’ skills 32 33 . Suggestions to optimize advanced endoscopy training in dedicated subspeciality training programs, requiring skills-based selection, were in line with previous research 34 . The main barriers to effective endoscopy supervision were lack of time and heavy workload, which is in line with a previous UK survey study 8 . Supervisors frequently had to perform other tasks during endoscopy supervision of residents, which may restrict supervisor availability, flexibility, and quality of supervision. Organizational commitment and support and having a set place and a regular time slot for supervision may help to overcome these barriers 8 23 . We propose that supervision should be seen as an organizational priority, enabling supervisors to focus on teaching. In addition, standardization of supervision practices is recommended, including sufficient time for supervision before and after an endoscopy training session.

A strength of this study is the mixed-methods design, which allowed for both an overview of the current endoscopy training practice and in-depth exploration of desired best practices. The nationwide design and high rate of response to the survey, with respondents from all teaching hospitals, is another strength. We acknowledge the following limitations. First, all interviews were conducted by a gastroenterology resident. Although being an insider had logistical advantages and was helpful for understanding participants’ views, it may also have affected collection and interpretation of the interview data 35 . Therefore, we built a research team with team members from within (AL,RM,WV) and outside (AD,JP,PB) the researched context. Second, for the semistructured interviews, we purposefully invited (associate) PDs, whose views on endoscopy supervision may differ from that of other endoscopy supervisors. Finally, our study was limited to gastrointestinal endoscopy training in the Netherlands, potentially limiting its international generalizability.

## Conclusions

In conclusion, this mixed-methods study found considerable variability in endoscopy training practices between teaching hospitals. Best practices were identified with respect to supervisors, residents, and context. Formal education about endoscopy teaching, promotion of resident SRL, and standardization of endoscopy training programs and supervision practices has the potential to improve future endoscopy training. Future studies should be conducted to evaluate the impact of formal education about and standardization of endoscopy teaching in endoscopy training practice.

## Supplementary Material

Supplementary Material Zusatzmaterial
