# Supplementary material for: Advances and challenges in endoscopy training: A mixed methods study among endoscopy trainers in the Netherlands
Source: Endosc Int Open. 2024 Sep 9;12(9):E1006–14. doi: 10.1055/a-2370-5812 (PMC11586648; doi:10.1055/a-2370-5812)
Supplement: Supplementary file 1 — Supplementary Material [file 10-1055-a-2370-5812_23765512.pdf]

Supplementary material

Supplementary materials

Appendix 1

Survey questions for endoscopy trainers

Demographic variables

1. Gender (m/f)
2. Age, … years (free text)
3. Name of the current teaching hospital (dropdown list)

Endoscopy training program in the teaching hospital

4. In my teaching hospital, the initial period under direct supervision (EPA level 2) is predefined and independent of the skills development of residents (yes/no/don't know).
5. If applicable: In my teaching hospital, the initial period under direct supervision (EPA level 2) is predefined in (time/number of performed procedures/other).
6. If applicable: The predefined period under direct supervision (EPA level 2) is approximately … weeks (free text).
7. If applicable: The number of performed procedures during the period under direct supervision (EPA level 2) is approximately … (free text).
8. If applicable: Specify other (free text).
9. If applicable: Which criteria are used to determine the transition from direct supervision (EPA level 2) to indirect supervision (EPA level 3) in your teaching hospital? (free text).
10. I am satisfied with the way the endoscopy training program is organized in my teaching hospital (5-point Likert scale).

Endoscopy supervision in the teaching hospital

11. In my teaching hospital, I supervise residents in the endoscopy room (yes/no).
12. If applicable: The number of years I supervise residents in the endoscopy room is approximately … (free text).
13. If applicable: The number of half-days a week I supervise residents in the endoscopy room is approximately … (free text).

Supplementary material

14. If applicable: The number of residents I supervise at the same time is approximately … (free text).
15. If applicable: In my teaching hospital, I supervise novice residents who perform endoscopic procedures under direct supervision (EPA level 2) (yes/no).
16. If applicable: In my teaching hospital, I supervise more experienced endoscopists who perform endoscopic procedures under indirect supervision (EPA level 3 or 4) (yes/no).
17. If applicable: The supervision of novice residents (EPA level 2) differs from the supervision of more experienced residents (EPA level 3 or 4) (yes/no).
18. If applicable: Explain the main differences in endoscopy supervision of novice and more experienced residents (free text).
19. If applicable: I have my own endoscopy program in parallel with the endoscopy supervision of more experienced residents (EPA level 3 or 4) (yes/no).
20. If applicable: I feel sufficiently competent in my role as endoscopy supervisor (5-point Likert scale).
21. If applicable: Endoscopy trainers in my teaching hospital provide uniform endoscopy supervision (5-point Likert scale).
22. If applicable: Before the start of an endoscopy supervision program under direct supervision (EPA level 2), I discuss the patients on the list with the resident (5-point Likert scale).
23. If applicable: Before the start of an endoscopy supervision program under indirect supervision (EPA level 3 or 4), I discuss the patients on the list with the resident (5-point Likert scale).
24. If applicable: After an endoscopy supervision program, I debrief the patients on the list with the resident (5-point Likert scale).
25. If applicable: Before the start of an endoscopy supervision program, I set learning objectives together with the resident (5-point Likert scale).

Training-the-endoscopy-trainer

26. I have received formal training on endoscopy teaching (yes/no).
27. If applicable: Specify the type of training and where this training took place (free text).
28. If applicable: The formal training on endoscopy teaching was useful and helped me to become a better endoscopy supervisor (5-point Likert scale).

Supplementary material

29. Formal training on endoscopy teaching should be mandatory for endoscopy supervisors (5-point Likert scale).

Open-ended questions

30. Positive aspects of the endoscopy training program in my teaching hospital are … (free text).
31. Areas for improvement of the endoscopy training program in my teaching hospital are … (free text).
32. Empty field to enter additional remarks: … (free text).

Supplementary material

Appendix 2

Semi-structured interview guide

- Describe the current endoscopy training program of residents in your teaching hospital.
- Describe future best practices regarding the endoscopy training program of residents.
- Describe the current endoscopy supervision practice of residents in your teaching hospital.
- Describe future best practices regarding the endoscopy supervision of residents.
- Are you satisfied with the endoscopy training of residents in your teaching hospital? Specify why you are (not) satisfied.
- Describe an endoscopy supervision program when you were satisfied with your role as endoscopy supervisor.
- Describe an endoscopy supervision program when you were less satisfied with your role as endoscopy supervisor.
- Our survey results indicated lack of uniformity in endoscopy teaching methods between different gastroenterologists in the same teaching hospital. What is your opinion on this lack of uniformity?
